# ‘Multi-omic’ data analysis using O-miner

**DOI:** 10.1093/bib/bbx080

**Published:** 2017-08-04

**Authors:** Ajanthah Sangaralingam, Abu Z Dayem Ullah, Jacek Marzec, Emanuela Gadaleta, Ai Nagano, Helen Ross-Adams, Jun Wang, Nicholas R Lemoine, Claude Chelala

**Affiliations:** 1 Barts Cancer Institute, Queen Mary University of London; 2 Barts Cancer Institute, co-Lead of the Computational Biology Centre at the Life Science Initiative, Queen Mary University of London

**Keywords:** multi-omics, sequencing, data analysis, data integration, O-miner

## Abstract

Innovations in -omics technologies have driven advances in biomedical research. However, integrating and analysing the large volumes of data generated from different high-throughput -omics technologies remain a significant challenge to basic and clinical scientists without bioinformatics skills or access to bioinformatics support. To address this demand, we have significantly updated our previous O-miner analytical suite, to incorporate several new features and data types to provide an efficient and easy-to-use Web tool for the automated analysis of data from ‘-omics’ technologies. Created from a biologist’s perspective, this tool allows for the automated analysis of large and complex transcriptomic, genomic and methylomic data sets, together with biological/clinical information, to identify significantly altered pathways and prioritize novel biomarkers/targets for biological validation. Our resource can be used to analyse both in-house data and the huge amount of publicly available information from array and sequencing platforms. Multiple data sets can be easily combined, allowing for meta-analyses. Here, we describe the analytical pipelines currently available in O-miner and present examples of use to demonstrate its utility and relevance in maximizing research output. O-miner Web server is free to use and is available at http://www.o-miner.org.

## Introduction

Large amounts of data have been generated from high-throughput profiling platforms. Public repositories, such as the Gene Expression Omnibus (GEO) [1], ArrayExpress [2], Sequence Read Archive (SRA) [3] and the European Genome-phenome Archive (EGA) [4], contain thousands of profiles from transcriptomic, genomic and methylation platforms across many experimental conditions and sample types. The exponential growth in the number of available data sets has created many challenges to data analysis and integration. The Bioconductor project (www.bioconductor.org) provides a vast array of open-source packages within the R programming environment for the analysis of data from both array and sequencing platforms. A typical pipeline for the analysis of array/sequencing data will require the use of several R packages and substantial coding skills to navigate between inputs and outputs from one package to another. This is not a simple task for those without any programming or data analysis experience, making the process both difficult and time-consuming. To make these workflows more accessible and useful to biologists and clinicians, it is necessary to create easy-to-use online tools to perform the required bioinformatic and statistical analyses. While several online tools are available for the analysis of data from transcriptomic and genomic experiments such as Babelomics 5.0, ArrayMining, Patchwork, wANNOVAR and the Tumor Aberration Prediction Suite [5–9], most cover just one type of data, or require bioinformatics expertise to interpret the data. Consequently, the need arises for comprehensive and easy-to-use online bioinformatics tools that are able to process raw profiles either individually or as a meta-analysis, while alleviating the need for researchers to invest time and effort in setting up the necessary computational infrastructure.

To satisfy this, we developed O-miner [10] as a solution for the analysis and exploitation of data. All analytical pipelines are designed to run in the R statistical environment and use well-established statistical methods from Bioconductor packages. Since its first publication in 2012, we have greatly improved O-miner by adding a number of analytical and graphical features to increase functionality and to improve the usability of our software by the scientific community. Here, we present an overview of O-miner and focus on its enhanced workflows and computational features. We also illustrate examples of use for the analysis of multiple transcriptomics data sets from breast cancer (BC) studies and the post-processing of RNA sequencing (RNA-Seq) data from prostate cancer (PCa) samples to extract biologically meaningful results. Feedback from the user community has resulted in many significant additions and improvements to the O-miner query system, analytical workflows and output layers since the first release. Table 1 summarizes the analytical workflows with corresponding input and output features that are currently supported in O-miner. Details regarding each of the analytical workflows are available from our comprehensive online user guide (http://o-miner.org/guide_2.0.html).

**Table 1:** Comparison of workflows and features between O-miner version 1.0 and version 2.0

	Pipeline	Feature	O-miner v1.0	O-miner v2.0
Supported platforms	Transcriptomics	Affymetrix Expression Array (Human Genome)	✓	Features added to this pipeline (see below)
		Affymetrix Expression Array (Mouse Genome)	✗	✓
		Illumina Expression Array (Human Genome)	✗	✓
		Illumina Expression Array (Mouse Genome)	✗	✓
		Affymetrix microRNA Array (Human Genome)	✗	✓
		Affymetrix Exon Array (Human Genome)	✗	✓
		RNA-Seq (post-processing)	✗	✓
	Genomics	Affymetrix SNP Array (Human Genome)	✓	Simplified
	Methylation	IlluminaMethylation Array (Human Genome)	✗	✓
Input parameters	Transcriptomics	Data type	Raw CEL file, normalized/filtered	
		Data source	User-defined, GEO repository	Automated suggestion for phenotype annotation from GEO data set
		Analysis type	Paired, unpaired	
		Provision for technical replicate	✓	
		Provision for batch effect correction	✗	✓
		Provision for survival data	✗	✓
		Provision for estimate tumour purity	✗	✓
		Provision for uploading target matrix	✗	✓
	Genomics	Analytical pipeline	CBS	ASCAT, genome sequencing (post-processing)
		Data type	Raw CEL file, normalized, segmented, binary coded	
		Data source	User-defined, GEO repository	
		Analysis type	Paired, unpaired	
		Baseline	User-defined, HapMap	
		Provision for uploading target matrix	✗	✓
	Methylomics	Data type	✗	Raw IDAT file, normalized
		Data source	✗	User-defined, GEO repository
		Analysis type	✗	Paired, unpaired
		Provision for technical replicate	✗	✓
		Provision for batch effect correction	✗	✓
		Provision for uploading target matrix	✗	✓
		QC	ArrayMvout, ArrayQualityMetrics	LUMI (Illumina array)
Analysis parameters	Transcriptomics	Normalization	RMA, GCRMA, TRMA	RSN, SSN, VSN, Quantile (Illumina array)
		Filter method	IQR, SD, intensity	
		Differential expression method	LIMMA	Edge R (RNA-Seq)
		Adjustment method	BH, FDR, BY, Holm	
		Provision for *P*-value threshold	Yes	
		Provision for fold-change threshold	Yes	
		Gene annotation system	RefSeq, Ensembl, UCSC, Vega	
	Genomics	Miscellaneous	miRNA, Cytoband, conserved TFBS	
		Minimial common region finder algorithm	CGHRegions	
		Provision for defining CNA region	Yes	
		QC	✗	ChAMP
	Methylomics	Normalization	✗	BMIQ, SWAN, PBC
		Filter method	✗	IQR, SD, intensity
		Differential methylation method	✗	LIMMA
		Adjustment method	✗	BH, FDR, BY, Holm
		Provision for *P*-value threshold	✗	Yes
		Provision for fold-change threshold	✗	Yes
		QC	ArrayMvout report, ArrayQualityMetrics report, Cluster plot	LUMI report (Illumina array), tumour purity report
Output	Transcriptomics	Differential expression	Gene level	Transcript, exon, splice level (Affymetrix Exon array)
		Miscellaneous	GO, Venn diagram, Expression plot	Survival plot, correlation tables
		QC	Density plot, cluster plot	
	Genomics	Copy number alteration	Gain, Loss	Copy neutral LOH (ASCAT), copy number from genome-sequencing data
		Visualization	CNA regions (sample and group level), MCR (group level)	
		QC	ArrayMvout report, ArrayQualityMetrics report, Cluster plot	Output from ASCAT algorithm
	Methylomics	Differential methylation	✗	CpG island level
		Miscellaneous	✗	GO, Venn diagram, methylation plot, correlation table

*Note*: Workflows and features available in O-miner version 1.0 are compared with O-miner version 2.0

## O-miner features

### Query submission architecture and data source

The basic O-miner request–response internal architecture remains the same. Following the provision of a project name and an email address (optional), users can upload data to O-miner via the user interface either as a zipped archive (of raw CEL files or a normalized data matrix from in-house or public data) or via the input of valid GEO GSE accession number(s). Users are then presented with a tabular form, where they need to assign biological groups to the uploaded data. While previously completed online, the current version of O-miner facilitates the assignment process by offering the option to upload a text file containing the relevant information for each sample. Analysis of individual and multiple data sets from the GEO has also been improved. In addition, extraction of biological information regarding the samples from GEO has been automated, bypassing the requirement for any manual inputting of data. Up to five user-defined data sets from GEO (using GSE accession numbers) can be automatically uploaded to O-miner.

An updated Perl CGI pipeline connects the data submitted through the front-end Web interface to the back-end workflows implemented in R. The results are displayed back to the users, once each of the analytical steps has been completed. Users are notified via email with the URL from where the results can be viewed. The results generated by O-miner are accessible online and are available for download without restrictions. Uploaded data sets and computed results are stored on our system for a period of 2 weeks.

### Analytical workflows: updates and additions

The first version of O-miner was composed of two analytical domains. These are genomics and transcriptomics, where the latter contained just one workflow for the analysis of data from the Affymetrix GeneChip Human Genome series only. The updated version includes an improved workflow for transcriptomic data and allows the analysis of data from the Affymetrix GeneChip Mouse Genome. Furthermore, new workflows have been added for the analysis of data from the Illumina expression array, Affymetrix exon array, Affymetrix microRNA (miRNA) array and the downstream processing of data from RNA-Seq experiments.

Previously, the genomics layer contained one workflow for the analysis of data from Affymetrix SNP arrays, which offered the use of several different algorithms for the segmentation stage of the analysis. However, in practice, when several of these are chosen, the analysis became both time-consuming and computationally expensive. The genomics layer has been improved. We have now implemented an improved version of the workflow using just one segmentation model circular binary segmentation (CBS), which is widely used in copy number analysis. This has simplified the process for users and limited the time and computational burden of analyses. We have also added two new workflows to the genomics section: the allele-specific copy number analysis of tumour (ASCAT) workflow and the genome sequencing (post-processing) workflow to estimate copy numbers from whole-exome sequencing (WES) and whole-genome sequencing (WGS) data.

A third, analytical layer for the analysis of data from methylation arrays has also been implemented. A list of data types and -omics platforms currently supported by O-miner is provided in Table 2.

**Table 2:** Platforms and data types supported by O-miner

Workflow	Data	Manufacturer	Platform
Transcriptomics	R, N	Affymetrix	miRNA 2.0
			miRNA 3.0
			GeneChip Human Exon 1.0ST
			GeneChip Human Gene 1.1ST & 2.0ST
			GeneChip Human Genome Array U133 Plus 2.0
			GeneChip Human Genome Array U133 set
			GeneChip Human Genome Array U95 set
			GeneChip Mouse Genome 430 2.0
	N, U	Illumina	HumanHT-12 v3
			Human HT-12 v4
			MouseRef-8 v2.0
		Multiple	RNA-Seq (post-processing only)
Genomics: CBS	R, N, S, B	Affymetrix	10K
			50K Xba
			50K Hind
			100K
			250K Sty
			250K Nsp
			500K
			Genome-Wide Human 5.0 SNP array
			Genome-Wide Human 6.0 SNP array
Genomics: ASCAT (cancer-specific)	R	Affymetrix	50K Xba
			50K Hind
			100K
			250K Sty
			250K Nsp
			500K
			Genome-Wide Human 5.0 SNP array
			Genome-Wide Human 6.0 array
Genomics: Sequencing	P	Multiple	Genome-sequencing (post-processing only)
Methylation	R, N	Illumina	Infinium HumanMethylation 27K BeadChip
			Infinium HumanMethylation 450K BeadChip

Code: R: raw; N: normalized; U: unnormalized; S: segmented; B: binary coded; P: processed.

*Note*: O-miner supports the analysis of pre-processed data from RNA-Seq experiments and genomic sequencing data; raw/processed data files generated using Affymetrix and Illumina transcriptomic and genomic arrays; and raw/processed data files from the Illumina Infinium methylation platform.

#### Transcriptomics

The core workflow comprised the following steps: quality control (QC), normalization, filtering, differential expression analysis and the identification of statistically significant gene ontology (GO) terms. For each of the new platforms added to O-miner, the relevant functions to perform these steps have been implemented. In addition, we have increased the functionality of the existing Affymetrix transcriptomics pipeline by adding the option of estimating tumour purity with the algorithm ESTIMATE (Estimation of Stromal and Immune cells in Malignant Tumours using Expression data) [11]. To increase the statistical robustness of meta-analyses, users now have the option to use the algorithm COMBAT [12] that eliminates batch effect(s) when integrating data from different studies from the same platform. We have also included an option for survival analysis.

**Figure 1:**
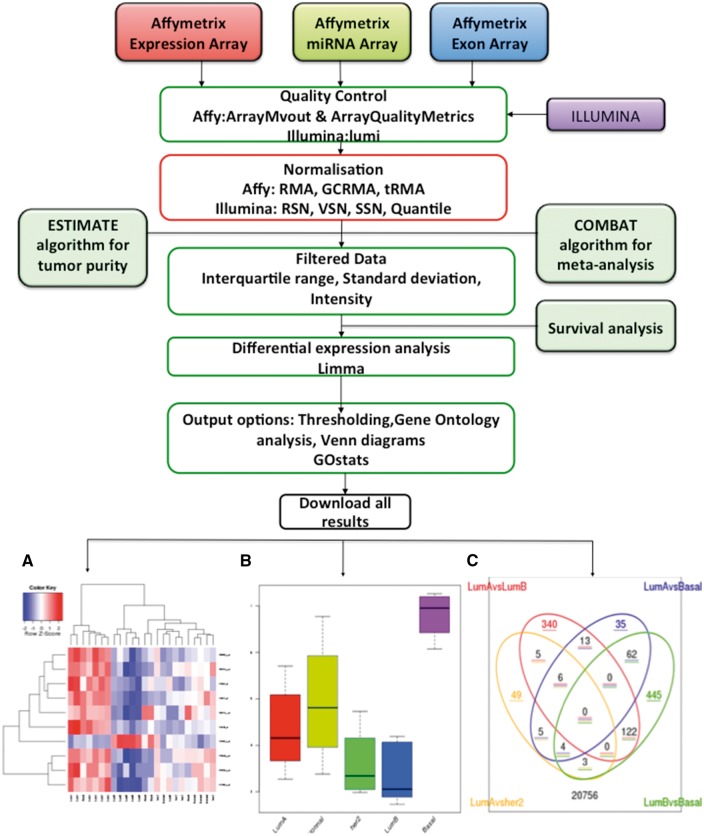
Transcriptomics workflow. O-miner takes as input raw array data (CEL files) from Affymetrix array-based platforms and either normalized/unnormalized data from Illumina expression arrays. QC is performed on data from raw CEL files. Data are then normalized and filtered to remove redundant probes. Users performing meta-analysis have the option to apply the COMBAT algorithm to correct for batch effects when combining data from different studies. Tumour purity can be estimated for Affymetrix data using the ESTIMATE algorithm. Survival analysis can be run for data from all of the array-based platforms. The normalized expression matrix is then subjected to differential expression analysis using LIMMA to identify significantly DEGs between biological groups. Optionally, GO terms that are statistically over- or under-represented are identified using GOstats, and Venn diagrams may be generated. Results are displayed online in expandable tabs and easy to download as text and excel files. (**A**) Heatmaps of the statistically significant DEGs identified for each of the comparisons are available to download. (**B**) A boxplot displaying the expression profiles across the biological conditions can be viewed. (**C**) A Venn diagram showing common and unique genes that are differentially expressed across the biological groups is displayed, if selected from the output options.

Results can be viewed from a single Web page, with data from each step of the analysis presented in a distinct tab (Figure 1). The QC assessment for raw .CEL files is implemented using the R package ArrayMvout [13]. These are presented as both a summarized report and as individual plots. ArrayMvout automatically excludes outliers from the analysis. Additional QC checks, from ArrayQualityMetrics [14], can be applied. Optionally, the COMBAT algorithm can be applied to a meta-analysis, which is usually performed after the normalization step. An estimated tumour purity report is also generated (for Affymetrix GeneChip Human Genome series only), if the ESTIMATE algorithm is run.

Normalized data matrices are derived using platform-specific normalization methods followed by a filtering step. An unsupervised hierarchical clustering algorithm is used on the normalized gene expression data to generate dendrograms to show similarity between samples. Filtering reduces the dimensionality of the data using one of the three following methods: interquartile range (IQR) (soft; intermediate; robust), intensity (25 or 50% of samples) or SD (up to the top 40% of the most variable probes). Differential expression analysis is then applied to the filtered matrix of normalized expression values using LIMMA [15] to identify significantly altered probes between the biological groups in the user-defined comparisons. For each comparison, the differentially expressed genes (DEGs), passing the user-imposed cut-offs of adjusted *P*-values and log fold-change, are presented in a tabular format as an annotated list or graphically as heatmap (Figure 1A). Users can view boxplots displaying the expression of DEGs across the predefined biological groups (Figure 1B). Optionally, Venn diagrams can be generated showing unique and overlapping genes that are differentially expressed in up to four biological comparisons (Figure 1C). The DEG lists can subsequently be used to identify significantly under- and over-represented GO terms using the R package GOstats [16], which includes ontologies relating to molecular function, biological process (BP) and cellular component. For data sets where survival covariates are supplied, three Kaplan–Meier (KM) plots are generated, to show 5, 10 and 15 years of survival rates across different risk groups [17].

The current version also has incorporated additional functionalities to interrogate the data. Previously, users could only visualize expression boxplots for their gene(s) of interest across the biological groups. Users now have the option to visualize the effect of those genes on survival as KM plots. A univariate model is applied to the survival data, and samples are assigned to risk groups based on the median dichotomization of mRNA expression intensities of the respective genes. Users can also identify genes that are co-expressed with their gene(s) of interest. The top 10 genes with the highest correlation, in terms of Pearson product-moment correlation coefficients (PPMCCs) and associated *P*-values, are presented in a table.

A number of default values are set against the various analysis parameters to assist non-advanced users: ArrayMvout is the default method for detecting outliers in the QC step; data are normalized using Robust Multi-array Average (RMA) and filtered using SD, where the top 40% of the most variable probes on the array are used for differential expression analysis; an adjusted *P*-value threshold of 0.05 and a log_2_ fold-change threshold of 2.0 are imposed to identify DEGs.

#### RNA-Seq post-processing

##### 

**Figure 2:**
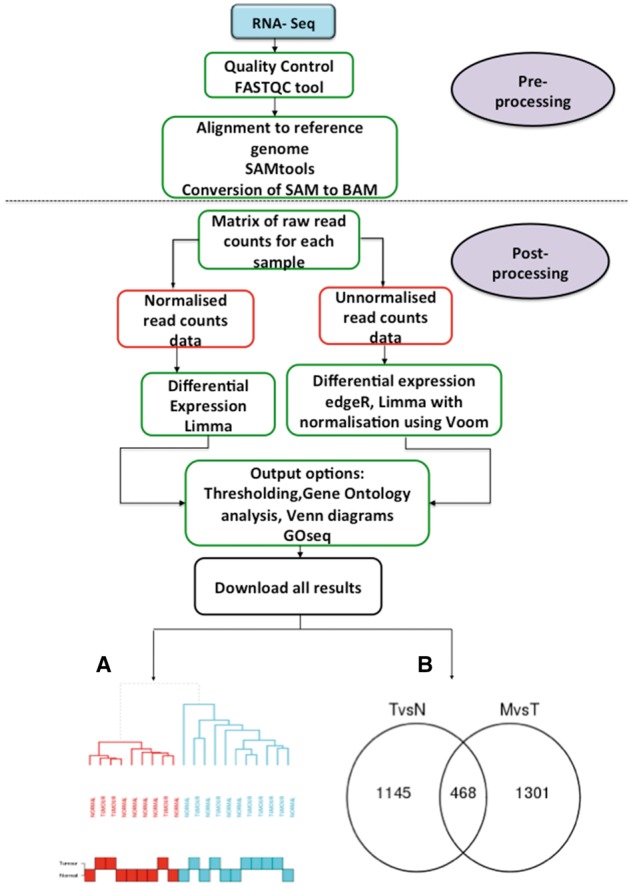
RNA-Seq post-processing workflow. O-miner provides a workflow for the post-processing of data from RNA-Seq experiments. After the pre-processing stage, comprising QC and alignment steps, a matrix of either raw read counts or RPKM values for each sample are submitted to O-miner. A choice of differential expression analysis methods is available—LIMMA for raw read counts and RPKM values, and edgeR for raw read counts. Like the transcriptomics workflow, users can then select the output options that they wish to implement. These include GO analysis and Venn diagrams. All the results are available as text and excel files and are available for download. The result options and presentation are identical to those generated by the transcriptomics workflow. (**A**) Unsupervised hierarchical clustering plot from raw read counts data, displaying similarity between gene expression profiles. (**B**) Venn diagram showing the number of unique and common DEGs between the biological groups.

Along with array-based transcriptomic data analysis, O-miner now also supports the post-processing of RNA-Seq data (Figure 2). Analytical steps covered by this pipeline include differential expression, annotation with Ensembl Gene IDs (if data are not already annotated) and the identification of statistically significant GO terms using the R package GOseq. Users can provide raw read counts generated from HTSEQ [18] or Reads Per Kilobase per Million mapped reads (RPKM) values. They can choose between LIMMA and edgeR [19] as methods for differential expression analysis, when raw read counts are provided. However, only LIMMA is available, when a matrix of RPKM values is uploaded. LIMMA applies the voom [20] transformation to raw read counts data to generate log counts per million with associated precision weights to be used for differential expression analysis, whereas EgdeR applies a generalized linear model to the data to calculate differential expression.

Results are presented in the same format as those from the other transcriptomics workflow. Further details of results generated from this pipeline can be found in our ‘Examples of use’ section.

#### Genomics

**Figure 3:**
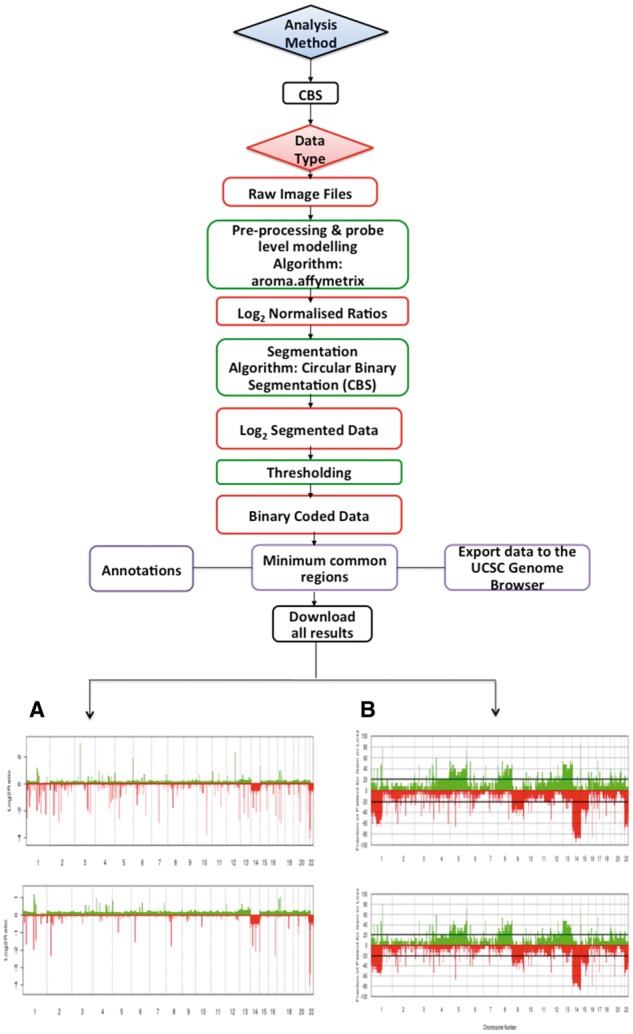
Workflow for CBS analysis. The CBS pipeline generates information about regions of gain and loss. Several steps comprise the CBS workflow, with the steps conducted being dependent on the input type. Raw image CEL files, log_2_ratios, segmented or binary coded data for a number of Affymetrix SNP arrays are used as input for the workflow. Aroma.affymetrix is applied to the raw CEL files to estimate copy numbers, data normalization and QC. Segmentation is applied using the CBS model. The quartile regression framework is applied to calculate the threshold used to call gains and losses. Regions of gain and loss are annotated from multiple sources. Minimal common regions can be generated using the CGHregions algorithm. (**A**) The results from each sample are displayed in expandable tabs. These tabs can be expanded further to obtain information about regions of loss and gain, with all findings available to download as an excel file by clicking on the ‘xls’ link. Log_2_ratio plots based on filtered and unfiltered data are displayed and can be downloaded as PDFs by clicking on the ‘PDF’ icon. (**B**). For each of the biological groups, frequency plots from both filtered and unfiltered data can be viewed either across all chromosomes or for individual chromosomes. All the filtered frequency plots are available for download as a zipped file by clicking on the arrow on the right-hand side of the window displaying chromosome number. Unfiltered frequency plots can be downloaded as PDFs by clicking on the ‘PDF’ icon. Results shown are from the analysis of data set GSE42525.

**Figure 4:**
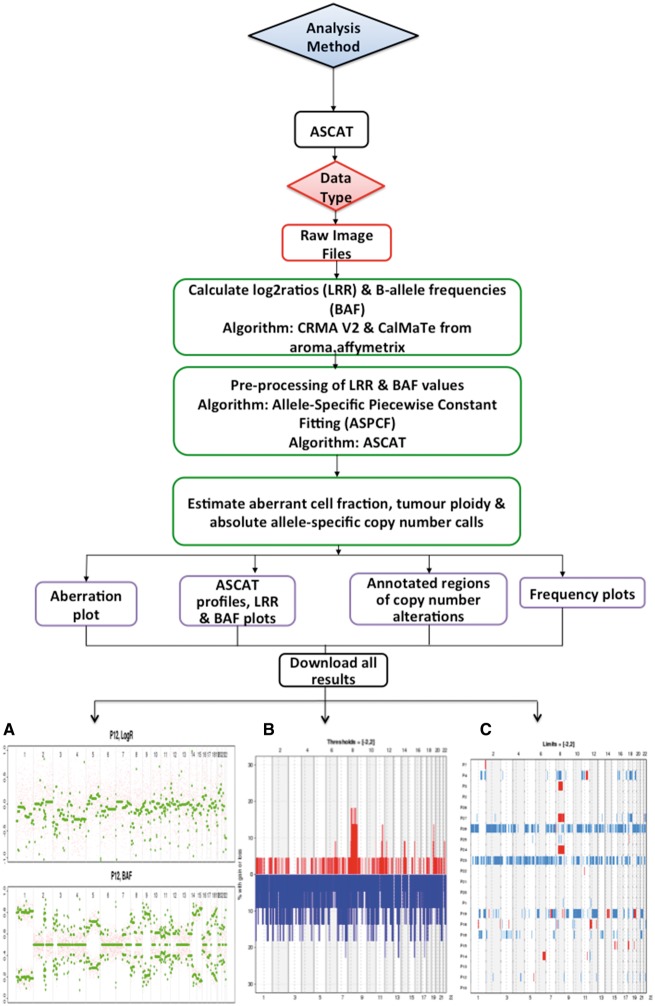
Workflow for ASCAT analysis. Raw data files are accepted as input. Log_2_ratios (LRR) and BAFs are calculated using the the R package CalMaTe. These are fitted to an ASPCF model. The ASCAT algorithm is used to estimate aberrant cell fraction, tumour ploidy and absolute allele-specific copy number calls. The results presented are from the analysis of the GSE7130 data set. (**A**) Raw LRR and BAF plots generated from ASCAT are shown for each sample. (**B**) Frequency plots of CNAs are also displayed for each biological group, with all frequency plots available for download as a zipped file. Frequency plots are shown across all the chromosomes and also for each individual chromosome. (**C**) Aberration plots are generated, showing regions of gain (red) and loss (blue) across each of the samples in the data set.

O-miner offers two analytical workflows, CBS and ASCAT [21], for the analysis of copy number data generated on Affymetrix array platforms (Table 1, Figures 3 and 4). Both workflows can conduct a complete genomic analysis from raw data files. In addition, the CBS workflow can also perform analysis from multiple entry points with processed data as input. A third workflow is offered for the post-processing of sequencing data, which estimates copy numbers from pre-processed WES and WGS data using the ASCAT algorithm (Figure 4). Both CBS and ASCAT workflows have common analytical steps, including background correction, allelic crosstalk calibration, nucleotide-position probe sequence effects normalization, probe-level summarization using robust average (SNP 5.0 and SNP 6.0 arrays) or log-additive model (10K, 100K and 500K arrays), polymerase chain reaction fragment-length effects normalization and calculation of raw copy number estimates (log_2_ratios) relative to the chosen reference. QC cluster or aberration density plots are generated for each platform using the R package aroma.affymetrix [22]. Default parameters are set to facilitate the analytical process for non-advanced users. These include a log_2_ratio (copy number ratio) threshold of 0.2; a minimum number of 15 consecutive SNPs to define regions of copy number aberration (CNA) as a gain/loss; and an observation percentage of 20% that sets the minimum number of samples for which a copy number event must be observed.

Similar to a transcriptomic analysis, results are viewable from a single Web page, with data from the QC step and sample-specific CNA regions as well as recurrent CNA regions across groups presented in distinct tabs (Figure 3). CNA regions are reported with corresponding physical and cytogenetic (optional) mapping information. Users can also customize the analyses by selecting to retrieve results from one or more data annotation systems, e.g. Refseq, Ensembl, UCSC and Vega, information on overlapping regulatory elements, such as miRNAs [23] or conserved transcription factor binding sites. Recurrent CNA regions are available in both tabular and graphical formats, and can be viewed either as summarized across all chromosomes simultaneously or individually for each chromosome. Such information can be valuable for identifying putative disease-causing genes [24].

#### Circular binary segmentation

##### 

Both paired (e.g. tumour–normal) and unpaired analyses are available when raw CEL files are provided. If paired normal/baseline samples are not available, a user-defined baseline may be selected. In this case, O-miner generates a pooled average from the unpaired normal samples, against which each of the tumour samples are compared. Alternatively, HapMap data can be used as a baseline. O-miner has pre-compiled raw HapMap data from four human populations: African YRI (originating from Yoruba in Ibadan, Nigeria), Japanese JPT (from Tokyo, Japan), Han Chinese CHB (from Beijing, China) and European CEU (from Utah, USA with ancestry from Northern and Western Europe). The CBS workflow can also accept partially processed text files (normalized, segmented or binary) of log_2_ratios along with the biological source/state specified for each sample.

O-miner conducts unsupervised hierarchical clustering for each sample using the raw log_2_ratios. If users do not specify threshold values, then the log_2_ratio threshold is calculated based on the quantile distribution of segmented copy numbers. These thresholds are then applied to the data to call copy number gains and losses. Figures 3A and B and 4C illustrate various plots that O-miner generates based on filtered or unfiltered log_2_ratio data.

#### Allele-specific copy number analysis of tumour

##### 

The ASCAT workflow is useful to study diseases caused by somatic mutations (Figure 4). O-miner accepts only raw .CEL files for this workflow, where both paired and unpaired analyses are available. CalMaTe [25] is used to calculate the log_2_ratios and B-allele frequencies (BAFs) between samples. These values are then processed with the allele-specific piecewise constant fitting (ASPCF) algorithm. For each sample, ASCAT profiles (Figure 4A) are generated with aberrant cell fraction and tumour ploidy information. Absolute allele-specific copy number calls are estimated with annotated regions of gain, loss or copy neutral loss of heterozygosity (LOH) (Figure 4B). Frequency and aberration plots are also generated (Figure 4C), using the R package DNAcopy [26].

#### Estimation of copy number from WES and WGS data

##### 

The workflow to generate copy number information from WES and WGS data takes as input the pre-processed files containing summary information of reads from the comparison between each tumour–normal pair. Pre-processing steps to analyse these data comprise: QC, alignment and the generation of SNP and indel-variant genotyping information. Based on the numbers of reads supporting the reference and altered alleles for each variant between tumour and normal samples, log_2_ratios (LRR) and BAF values are calculated for each tumour–normal pair with the depth information normalized by dividing the depth of each variant by the median depth across all variants. These files are then used as input to the ASCAT algorithm to estimate copy number calls, annotate the regions of CNA as well as generating frequency and aberration plots.

#### Methylomics

**Figure 5:**
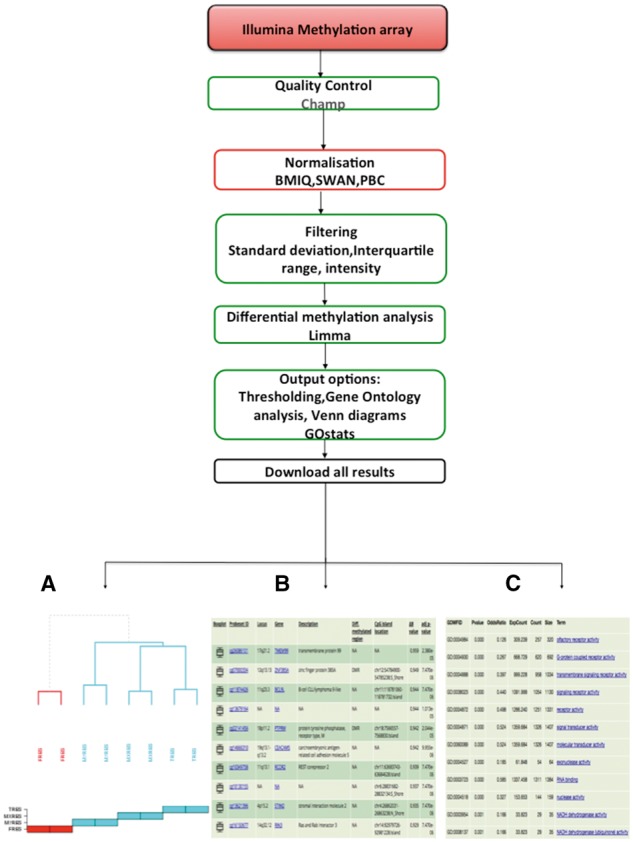
Methylation workflow. Raw (IDAT) files and normalized data from Illumina methylation array platforms are accepted as input to the methylation workflow. QC analysis is performed, using the Champ R package. One of the following normalization methods: BMIQ, SWAN and PBC can be chosen to normalize the data. After filtering of the normalized data, differentially methylated probes are identified using LIMMA, with user-defined thresholds for the delta beta value and adjusted *P*-values applied. Differentially methylated regions are annotated and users can choose to identify statistically significant GO terms from the list of differentially methylated probes. Results shown are from the analysis of data set GSE69118. (**A**) Sample quality, QC plots and cluster diagrams are presented. Sample quality displays a table showing the sample name and % of failed probes for each sample. QC plots consist of four plots that are available for display and download. These are raw density plot, normalized density plot, raw MDS plot and normalized MDS plot. Cluster diagram displays an unsupervised hierarchical cluster based on normalized methylation data. (**B**) Each comparison is displayed within an expandable tab alongside information about probeset ID, chromosomal location, HGNC symbol, gene description, whether the region is differentially methylated, location of CpG island, delta beta value and adjusted *P*-values. A boxplot, showing the difference in methylation values across biological groups, can be also viewed for each probeset ID. (**C**) Individual comparisons are displayed as separate tabs. Each of the probes reported as differentially methylated are mapped to GO terms, with those that were found to be statistically over- and under-represented listed in tabular format.

O-miner now offers an analytical workflow to analyse data generated on Illumina Infinium methylation arrays (Table 1, Figure 5). Similar to the transcriptomics workflow, the general structure of the methylomics workflow comprises key steps: QC, normalization and filtering, followed by differential methylation analysis and identification of statistically significant GO terms.

Input data can be provided as either raw files or normalized data. When raw files are provided, red and green IDAT files are uploaded for each sample. O-miner automatically combines the two files and extracts the sample name. Users then need to specify the biological state/source for the sample. Paired or unpaired analyses can be performed, and technical replicates can be flagged. QC analysis on the input data is performed using the R package ChAMP [27], which calculates the proportion of failed probes in each sample. A variety of quality assessment plots are also generated alongside a hierarchical cluster diagram (Figure 5A).

Raw data can be normalized into a matrix of beta values by selecting one of the following methods: BMIQ [28], SWAN [29] and PBC [30]. More information on each of these methods can be found in our online user guide. Normalized data generated by O-miner or user-provided normalized data are then used for filtering and subsequent differential methylation analysis to identify significantly altered probes. An annotated list of the differentially methylated regions is generated. Information is provided regarding the chromosomal location, corresponding hyper- or hypo-methylated genes and whether the probe region overlaps with a known region of differential methylation and/or a CpG island. Boxplots are also generated showing differences in methylation levels between biological groups. Users can also opt for generating Venn diagrams showing the number of probes exclusive or common to each of the biological groups.

GO terms found to be over- or under-represented amongst the differentially methylated probes can also be generated (Figure 5C). O-miner also allows users to interrogate the correlation between a probe of interest and others present on the array using the PMCC value.

### Elaborate documentation

To ensure that first-time users are able to understand all the available parameters for -omics analyses and customize their analysis accordingly, a comprehensive user guide describing each workflow is available online (http://o-miner.org/guide_2.0.html).

Additionally, an ‘Examples of use’ section is available (http://o-miner.org/examples_2.0.html), so that users can familiarize themselves with the analytical workflows, input file formats and structure of the output before analysing their own data. Examples are provided for each of the analytical pipelines with various parameter settings. The user interface also contains pop-up help buttons for selected fields to provide additional information.

## Examples of use

This section presents how the RNA-Seq and transcriptomics workflows can be applied to biological data to conduct independent and meta-analyses and to draw meaningful conclusions.

### Case study 1: Meta-analysis of BC transcriptomics data to investigate the relationship between triple-negative BC and basality

#### Background

BC is one of the leading causes of cancer-associated deaths among women worldwide. It is a heterogeneous disease exhibiting distinct histological and biological characteristics, diversity in clinical behaviour and variability in response to treatment. The ability to reliably classify and address these entities independently has important diagnostic, prognostic and therapeutic implications and is a major step towards a more personalized approach to the treatment of BC.

Seminal studies applying microarray-based technology to BC research demonstrated the phenotypic heterogeneity of BC to be accompanied by a parallelized diversity in transcriptomic profiles, and segregated BCs into five primary molecular subtypes—luminal A, luminal B, basal-like (BL), Her2+ and normal breast-like—each with distinct transcriptomic signatures.

The BL subtype represents 10–25% of all BC and is of particular interest to the cancer research community because of its aggressive clinical behaviour, lower overall survival relative to the other molecular subtypes and lack of targeted therapy. Clinically, this subtype is characterized by a high prevalence in premenopausal women, particularly those of African descent, large tumour size at diagnosis and specific metastatic patterns favouring dissemination to the brain and lungs. For Basal-Like Breast Cancer (BLBC) patients, the first-line treatment would be conventional chemotherapy.

This molecular subtype shares many features with the immunohistochemically defined triple-negative (TN) subgroup, which is characterized by a lack of clinically significant oestrogen receptor (ER), progesterone receptor (PR) and Her2 expression. The terms BLBC and Triple-Negative Breast Cancer (TNBC) have been used interchangeably in the past but, with discordance rates of ∼30% reported between tumours with the TN phenotype and those with the BL molecular phenotype, it is important that researchers address these definitions as distinct entities.

Triple-negative tumours assigned to the basal-like molecular subgroup (TNBL) have been associated with lower median age at presentation, higher pathological grade, increased tumour size and distinct differences in clinical outcome relative to TN tumours allocated to one of the other molecular subtypes (TNnonBL).

We used O-miner to conduct a multi-cohort meta-analysis of publicly available data to gain a deeper understanding of the relationship between TNBL and TNnonBL tumours.

#### Data

Subsets of BC samples profiled using the Affymetrix Human Genome U133 Plus 2.0 Array (GSE48390 [31], GSE21653 [32]) were downloaded from GEO.

The ER, PR and Her2 receptor status of each sample were defined by implementing functions within the MCLUST R library. The MCLUST algorithm was set to calculate the Bayesian information criterion for a two-component Gaussian distribution model. In addition, the PAM50 classifier was applied to determine the subtype calls of each sample. The TN samples were then isolated and grouped based on their basality, i.e. TNBL and TNnonBL.

These samples were uploaded to O-miner, and in-depth analyses of their transcriptomic profiles and survival characteristics were conducted.

#### Interpretation of output

**Figure 6:**
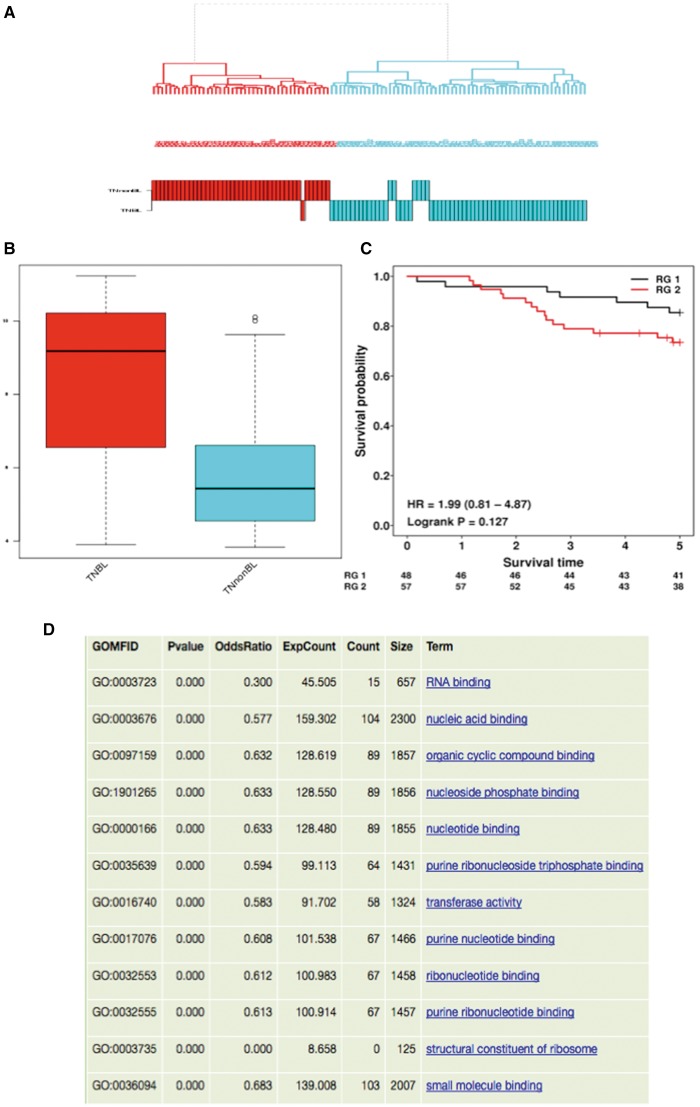
Application of the transcriptomics workflow for the multi-cohort analysis of BC data. *Data Collection:* A meta-analysis was conducted using O-miner to investigate the effect of basality on TN BCs. Two Affymetrix data sets GSE48390 and GSE21653 were downloaded using *GEO data set* as the data source option. The subset of samples defined as triple negative, were selected from the *File Organiser* window. *Analysis Parameters:* Once all the sample characteristics and survival covariates were provided, the raw data were normalized using RMA and filtered using SD (top 10%). Samples belonging to each of the data sets were specified and the COMBAT algorithm applied to adjust for batch effects. The resulting normalized matrix was subjected to differential expression and survival analyses. All the results are available and easy to download as text and excel files. *Results:* (**A**) Unsupervised hierarchical clustering of the gene expression profiles suggests that TNBL BCs are more similar to each other than to TNnonBL BCs. The cluster is annotated with the sample names and biological groups. Each biological group has its own colour. (**B**) The *GABRP* gene was reported differentially expressed between the two biological groups. The expression of *GABRP* between the TNBL and TNnonBL groups can be displayed by boxplots. (**C**) Survival, the 5-year KM survival plot suggests that the BLTN group has poorer overall survival relative to the BLnonTN group but this relationship is not significant (*P*>0.05). (**D**) Statistically significant GO terms between BLTN and BLnonTN groups are displayed, with hyperlinks to external resources provided.

Unsupervised hierarchical clustering to view the underlying structure of the data indicated that the gene expression profiles of TNBL BCs are more similar to each other than to those of TNnonBL BCs (Figure 6A).

The most DEGs between the two groups included *GABRP*, *ABCA8* and *DARC*, as well as various cytokeratins, which is in accordance with previous work. By focusing on a given gene, such as *GABRP*, O-miner presents its expression across the biological groups (Figure 6A and B). From this, we can examine the behaviour of the gene more clearly. For example, expression of *GABRP* is higher in TNBL tumours than in TNnonBL tumours, supporting previous research suggesting that *GABRP* is involved in the initiation and progression of BL tumours. Key GO terms identified as disrupted between TNBL and TNnonBL include antigen processing, cytokine activity and immune response, indicating that multiple immune processing pathways are affected in TNBL relative to TNnonBL [33] (Figure 6D).

The 5-year KM plot displays a trend for the Basal-Like Triple Negative (BLTN) group to have a poorer overall survival relative to BLnonTN; however, this relationship is not significant (*P* > 0.05) (Figure 6C) and disappears >10 and 15 years.

The results from this analysis suggest that TNBL and TNnonBL tumours exhibit unique transcriptomic profiles, with genes and pathways associated with immunological processes and cell signalling being reported as significantly disrupted between the two groups. This not only serves to confirm the ‘uniqueness’ of each group but also could indicate potential targets that warrant further investigation.

### Case study 2: Analysis of PCa sequencing data from The Cancer Genome Atlas

#### Background

PCa is the second most common male cancer and the fifth leading cause of cancer-related death in men [34]. It has long natural history and can initiate from disrupted prostate epithelium and progressively develop over many decades [35]. While PCa patients present remarkable diversity both in terms of pathology and clinical presentation [36], which can be partially explained by underlying genetic heterogeneity, in most cases, it is an indolent disease that is unlikely to ever become symptomatic during patients’ lifetime [37].

Many studies and collaborative efforts investigated PCa molecular make-up resulting in the identification of key alterations and associated molecular processes involved in the disease development. Therefore, we used PCa as proof of concept to test the robustness of the O-miner RNA-Seq post-processing pipeline for detecting genuine aberrantly expressed genes and also to search for novel gene associations with PCa.

#### Samples

**Figure 7:**
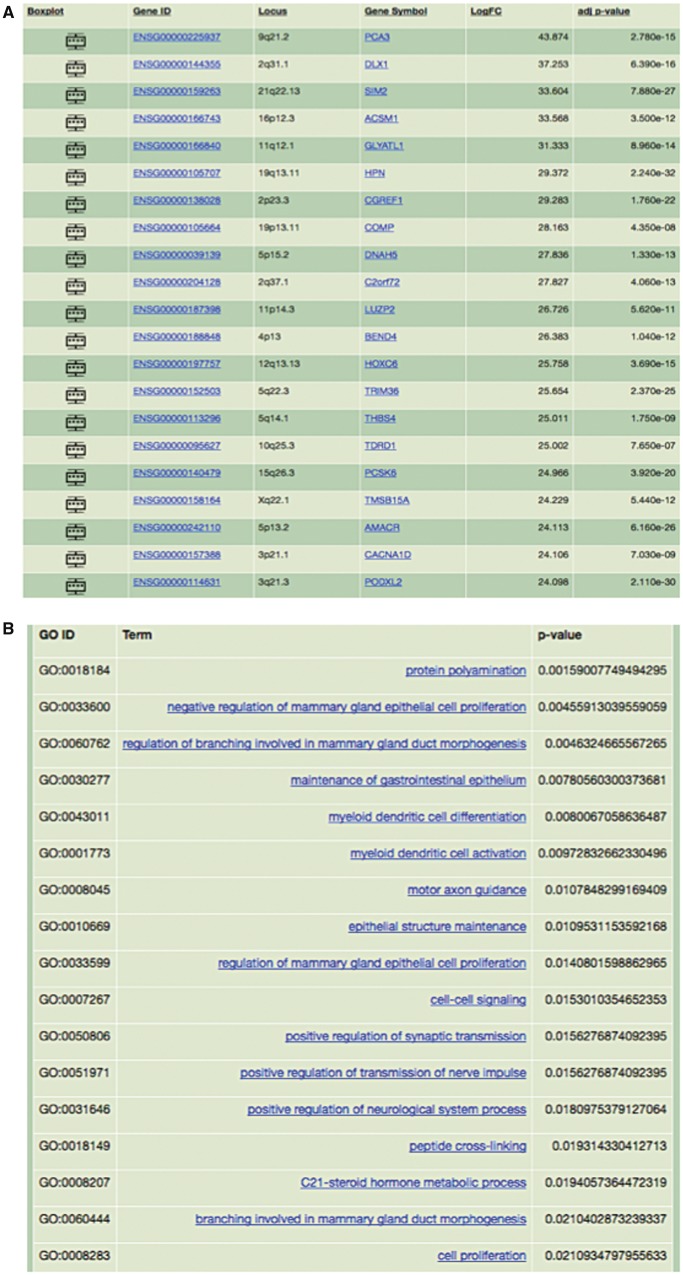
Application of O-miner to the analysis of PCa sequencing data. *Data collection*: Sequencing data from the TCGA PRAD project were downloaded and subjected to the O-miner RNA-Seq post-processing workflow. *Analysis parameters*: Following pre-processing of data (QC and alignment steps), a matrix of raw read counts was generated. The matrix of normalized read counts was submitted to O-miner. LIMMA was used to identify DEGs, and statistically significant GO terms were identified. Users can choose to generate Venn diagrams. All of the results are available as text and excel files and are available to download. *Results:* (**A**) Significantly DEGs are displayed together with Ensembl gene ID, chromosomal location, fold-change and adjusted *P*-values. (**B**) Results of GO analysis of DEGs are displayed in tabular format. Over- and under-represented GO terms are listed and GO IDs, *P*-values and GO term annotations are present.

RNA-Seq data from The Cancer Genome Atlas (TCGA) prostate adenocarcinoma (PRAD) project were downloaded and subjected to QC and alignments steps. These pre-processed data were uploaded to O-miner, and genes/GO terms differentially altered between PCa and normal samples were identified (Figure 7).

#### Interpretation of output

Several genes identified as differentially expressed by O-miner have previously been identified as PCa biomarkers including *PCA3*, *DLX1*, single-minded homolog 2 (*SIM2*), hepsin (*HPN*), *HOXC6*, *AMACR* (Figure 7A) as well as *MYC*, forkhead box O1 (*FOXO1*), *PTEN*, *RUNX2*, *MET*, *RB1*, *EGF*, *ERG*, *EZH2*, *FOXA1* and *SPINK*. The most significantly DEG *PCA3* encodes prostate cancer gene 3, which is a widely used urine biomarker for PCa [38]. *SIM2* encodes a transcription factor involved in PCa onset and progression [39]. *HPN* is one of the most consistently over-expressed genes in PCa and is associated with disease progression and metastasis [40]. Other top-ranked genes by O-miner and implicated in PCa are the homeobox genes *HOXC6* and *DLX1*, recently proposed as urine-based biomarkers for early disease diagnosis [41], as well as diagnostic marker *AMACR* [42], which encodes alpha-methylacyl-CoA. Moreover, we noted strong evidence for differential expression for *MYC*, a well-known oncogene [43] located in frequently amplified region 8q24 in PCa, and *FOXO1*, a key downstream effector of the tumour suppressor *PTEN* and critical gene in negative regulation of transcription factor *RUNX2*, which are also significantly deregulated according to results derived from O-miner.

Several top-ranked genes, which have not been linked with PCa, have been previously associated with other malignancies. For instance, *DNAH5* (dynein axonemal heavy chain 5) has an important role in the development of colorectal cancer [44], whereas *COMP* (cartilage oligomeric matrix protein) has been recently reported as a novel biomarker contributing to the severity of BC [45].

Results of GO analysis using DEGs are displayed (Figure 7B). Over- and under-represented GO terms are listed and GO IDs, *P*-values and GO term annotations are presented. Among the most enriched GO terms is steroid hormone metabolic processes (GO:0008207) associated with biosynthesis of cholesterol, whose increased level in the blood was previously linked with an increased risk of PCa and its aggressiveness [46]. Two other highly enriched GO terms (GO:0007411 and GO:0008045) are related to axon guidance, whose associated genes were previously linked with PCa [47] suggesting that these BPs may have important role in prostate tumourigenesis.

Several established PCa biomarkers were identified using O-miner. Moreover, many genes not previously reported in the context of PCa were identified from this analysis. Given the published evidence of their association with other malignancies, they are promising candidates for experimental validation and further exploration to characterize their functional role in PCa. This example illustrates that O-miner provides researchers with the tools required to conduct powerful analyses of publicly available sequencing data.

## Limitations and future directions

O-miner is an analytical suite that has filled an existing void for biologists to be able to perform increasingly complex -omics analyses without the need for bioinformatics support or a complex IT infrastructure.

Currently, a key limitation to O-miner is that it requires Next Generation Sequencing (NGS) inputs to be pre-processed. This means that the user needs to conduct QC and sequence alignment on sequencing data. Another limitation is the lack of workflows for Agilent arrays or non-Affymetrix copy number arrays starting from raw data. While O-miner provides links to gene ontologies, we appreciate that the utility of this resource would be greatly enhanced if links to pathway databases, such as KEGG [48] or Reactome [49], were also provided. Finally, O-miner is not yet able to integrate results from the different analytical layers, for example correlating gene expression with copy number information on matched samples.

The flexible design of the analytical modules comprising O-miner allows for the easy addition of further analytical processes to existing pipelines as well as new workflows. In that respect, our future plans include expanding on the analytical, computational and visualization capabilities of the tool and making it even more informative and useful for the research community.

## Availability and requirements

Project name: O-miner

Project home page: www.o-miner.org

Operating system(s): Platform independent; Standard WWW browser (Google Chrome, Safari and Mozilla Firefox).

Key Points

- O-miner provides a number of extensive analytical workflows for the analysis of high-throughput data.
- Data from transcriptomic arrays, pre-processed RNA- Seq, SNP array, WES and WGS data as well as methylation arrays can be analysed with ease.
- Raw data from GEO (multiple projects) or summarized data from TCGA can also be submitted and analysed.
- Results can be viewed online or downloaded in text, graphical and excel format.

## Notes

**Ajanthah Sangaralingam** is a Postdoctoral Research Fellow at Barts Cancer Institute, Queen Mary University of London. Her research interests lie in computational biology, software development and predictive analytics.

## Notes

**Abu Z Dayem Ullah** is a Postdoctoral Research Fellow at Barts Cancer Institute, Queen Mary University of London. His research interests lie in computational biology, software development and clinical informatics.

## Notes

**Jacek Marzec** is a Postdoctoral Research Fellow at Barts Cancer Institute, Queen Mary University of London. His research focuses on the development and application of algorithms to analyse high-throughput ‘multi-omic’ data in cancer studies.

## Notes

**Emanuela Gadaleta** is a Postdoctoral Research Fellow at Barts Cancer Institute, Queen Mary University of London. Her research focuses on computational biology and ‘multi-omic’ data integration in cancer studies.

## Notes

**Ai Nagano** is a Postdoctoral Research Fellow at Barts Cancer Institute, Queen Mary University of London. Her research focuses mainly on computational biology and biostatistics and their application to analyse high-throughput ‘multi-omic’ data.

## Notes

**Helen Ross-Adams** is a Postdoctoral Research Fellow at Barts Cancer Institute, Queen Mary University of London. Her research focuses mainly on molecular biology and its application to validate results from high-throughput ‘multi-omic’ data.

## Notes

**Jun Wang** is a Lecturer in Bioinformatics at Barts Cancer Institute, Queen Mary University of London. His research interests lie in computational biology, predictive analytics and non-coding genome data science.

## Notes

**Nicholas R. Lemoine** is the Director of Barts Cancer Institute, Queen Mary University of London. His research interests lie in molecular pathology, gene therapy and clinical informatics.

## Notes

**Claude Chelala** is a Professor of Bioinformatics at Barts Cancer Institute, co-Lead of the Computational Biology Centre at the Life Science Initiative, Queen Mary University of London. Her research focuses on ‘Big Data’ analysis and modelling for cancer diagnostics, prognostic and therapeutics.
